# Creating and testing a questionnaire to predict immediate and strong positive responders to spinal manipulative therapy for non-specific low back pain. A pilot study

**DOI:** 10.1186/s12998-023-00510-3

**Published:** 2023-09-26

**Authors:** Stanley Innes, Reece Granger, Jean Théroux

**Affiliations:** 1 School of Allied Health, Murdoch University, Perth, Australia; 2 Eastern Health, Adult Mental Health Wellbeing Program, Victoria, Australia

**Keywords:** Chiropractic, Therapeutic alliance, Patient expectations, Spinal manipulation

## Abstract

**Background:**

Many chiropractors use spinal manipulative techniques (SMT) to treat spinal pain. A recent Delphi study posited 18 items across five domains as predictors of patients experiencing non-specific low back pain most likely to experience a strong and immediate positive response to SMT. We sought to create a ‘pen and paper’ questionnaire that would measure these items and then pilot its use in a clinical setting to determine its ‘usability’ for a larger study. Knowing this information would inform a more efficacious use of SMT.

**Method:**

Of the 18 items identified in the Delphi study, 13 were deemed historical in nature and readily provided by the chiropractor and patient. A literature search revealed reliable and valid measures for two more items. The remaining three items were generated by creating descriptive questions matched to an appropriate Likert scale. A panel of six chiropractors who had used SMT for at least 7 years when treating non-specific low back pain was formed to evaluate the items for clarity and relevance. Ten Western Australian chiropractors were then recruited to pilot the questionnaire on ten consecutive patients with non-specific low back pain where SMT was used from March to June 2020. Ethics approval was obtained from Murdoch University.

**Results:**

COVID-19 restrictions impacted on practitioner recruitment and delayed the data collection. Of the intended 100 participants, only 63 could be recruited over a 3-month period from seven chiropractors. Time constraints forced the closure of the data collection. The measures of all predictor items demonstrated ceiling effects. Feedback from open-ended practitioner questions was minimal, suggesting an ease of use.

**Conclusion:**

The length of time and level of participation required to collect the calculated sample size was inadequate and suggested that incentivization may be required for a larger investigation. Significant ceiling effects were found and suggested that participants did so because of a positive bias toward chiropractic care and the use of SMT. The questionnaires in this pilot study require alternative measures and further validation before use in a larger study.

**Supplementary Information:**

The online version contains supplementary material available at 10.1186/s12998-023-00510-3.

## Background

Quality care is required from healthcare practitioners to assist in reducing the substantial financial impost of musculoskeletal pain [1, 2]. Manual therapists are part of the array of practitioners who treat and diagnose musculoskeletal disorders [3]. Many employ in their therapeutic arsenal high-velocity low amplitude spinal manipulation (SMT) [4]. Spinal manual therapy is often included in guidelines for care of spinal pain [5, 6]. Inherent in the notion of quality care is the efficacious use of any or all interventions.

Many theories have been studied trying to explain how SMT may relieve non-specific low back pain (LBP). While some studies looked at the bio-mechanical aspects [7–9], others have explored the psychosocial influences [10, 11]. To this end, a recent study sought the opinions of manual therapy practitioners (Delphi methodology) who were experts in SMT to the possible factors that would identify a patient who experiences a strong and instant positive response to SMT for non-specific LBP [12]. Nineteen practitioners with at least seven years of experience in using SMT as an intervention for LBP participated and included ten chiropractors, six physiotherapists/physical therapists, and three who were dual qualified. The participants identified 18 predictor items (Table 1). Several were not bio-mechanical and included the strength of the therapeutic alliance (TA) and the degree of mutual understanding of practitioner-patient expectations and goals for care [12]. These predictor items aligned with the contemporary healthcare framework that emphasises patient-centred care [13]. It was suggested that these predictors be grouped into five domains, namely patient factors, practitioner factors, specific patient signs and symptoms, a measure of fear-avoidance beliefs, and the presence of an audible release following SMT.

**Table 1:** Final 18 predictor items placed in 5 Domains ranked by mean score and sum

**Patient factors**
1. Patient history of a good response to previous SMT
2. Patient has trust and high confidence in the practitioner
3. Patient susceptible to placebo effect
4. Patient has a comprehensive understanding of condition
**Practitioner factors**
5. Good patient-practitioner relationship
6. Professional opinion of health status - excellent/ very good
7. Practitioner understanding of patient expectations and goals
8. Professional opinion of health status – good
**Signs and symptoms of NSLBP presentation**
9. Duration of symptoms < 16 Days
10. Pain improves with exercise, but not rest
11. No symptoms in the lower extremities
12. Patient has an acute condition (< 14 days)
13. No symptoms distal to the knee
14. Decreased active range of motion
15. Decreased passive range of motion
16. Close reproduction of symptoms on spinal springing and/or end range loading
**An instrument of measurement (FABQ)**
17. Fear Avoidance Beliefs Questionnaire work scale
**The presence of an audible release following SMT**
18. The production of an audible release (cavitation) at the moment of thrust

To assess these items for reliability and validity, as well as identify potential problems when applied in clinical settings, these 18 predictor items needed to be converted into a questionnaire.

The authors in this current study were two chiropractic academics and a chiropractic student researcher with considerable knowledge of the regional chiropractic community. Also, thirteen of the original Delphi study participants identified as being qualified chiropractors. Consequently, we chose to approach local chiropractors to better facilitate this student research project.

Thus, the aims of this study were twofold.

1. To create an actionable questionnaire from the identified 18 predictor items.
2. To pilot the resultant questionnaire for its “usability” for identifying strong and instantaneous responders to SMT for non-specific LBP within a chiropractic care context.

## Method

**Fig. 1:**
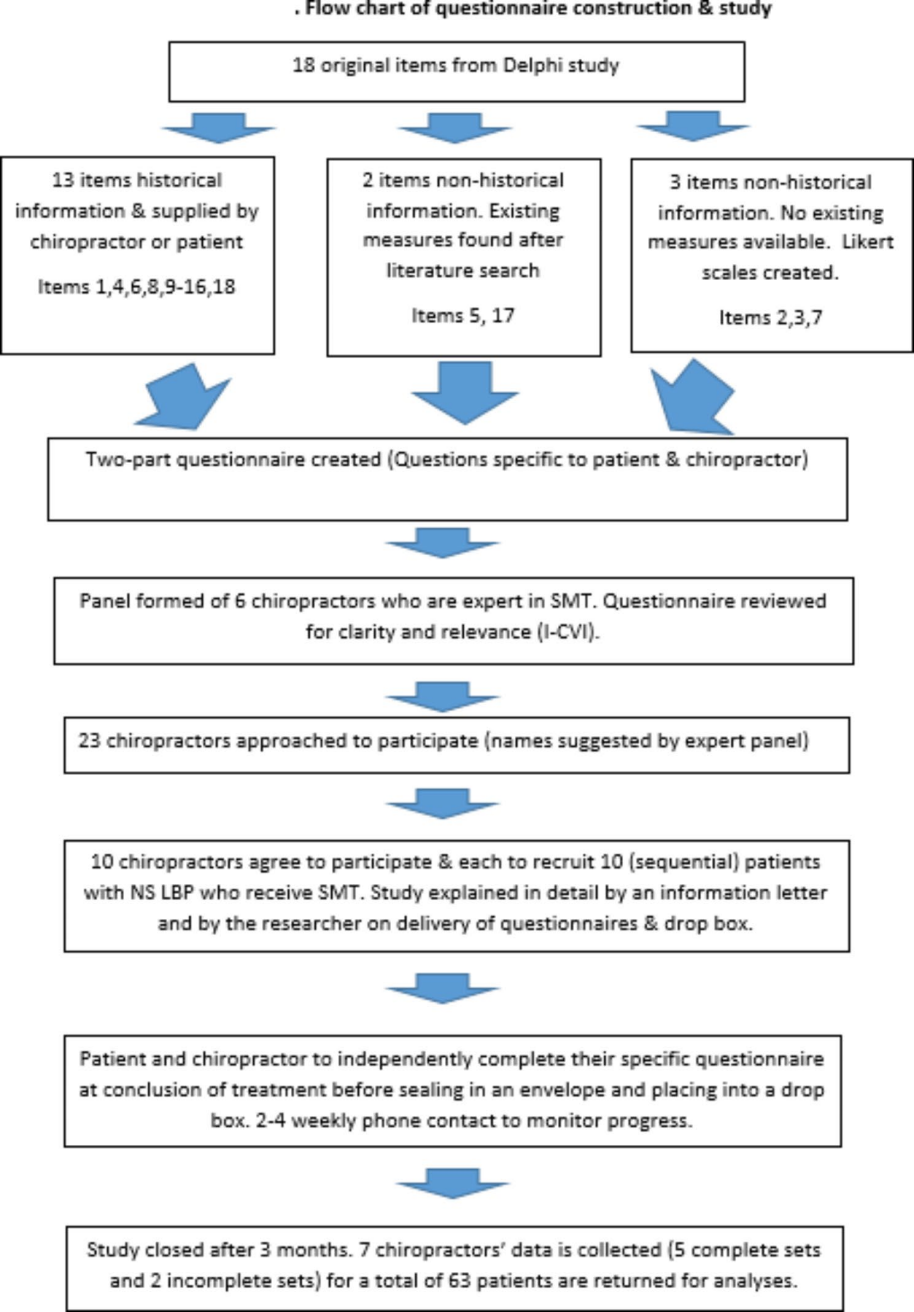
Flow chart of questionnaire construction & study. (*Abbreviations Fig. 1: SMT = spinal manipulative therapy. I-CVI: item content validity index. NS LBP: Non-specific low back pain*)

This cross-sectional and pragmatic study sought to create a ‘pen-and-paper’ self-administered questionnaire of the 18 predictor items identified in the Delphi study [12]. Measures were created for each predictor item and reviewed by a focus group for clarity and relevance. A pilot study was then conducted to test whether the procedures (sampling, data collection, analysis) were feasible or susceptible to bias. This process is outlined in Fig. 1. Ethics approval was granted by Murdoch University Human Research and Ethics Committee (2020/152).

### Questionnaire creation

Of the 18 items identified in the Delphi study [12], 13 were considered part of the patient history and could be provided by the chiropractor or patient. These were:

- Patient history of a good response to previous SMT (item 1).
- Patient has a comprehensive understanding of condition (item 4).
- Chiropractors’ opinion of patient health status as either good (item 8), very good, or excellent (item 6).
- Duration of symptoms < 16 days (item 9).
- Pain improves with exercise but not rest (item 10).
- Patient has an acute condition of < 14 days (item 12).
- No symptoms in the lower extremities (item 11) or distal to the knee (item 13).
- Decreased active and/or passive range of motion (items 14 and 15).
- Close reproduction of symptoms on spinal springing (item 16).
- An audible release following SMT (item 18).

Five items were deemed not part of the patient’s history and required either an inventory or rating scale. These were:

- Patient has trust and high confidence in the practitioner (item 2).
- Patient susceptible to placebo effect (item 3).
- Good patient-practitioner relationship (item 5).
- Chiropractors’ understanding of patient expectations and goals (item 7).
- Fear Avoidance Beliefs Questionnaire - work scale (FABQ-W) (item 17).

For these items (Items 2, 3, 5, 7, 17), a literature search was conducted to see if previous research had produced any reliable and/or valid measures. A suitable measure was found for “Good Patient-Practitioner Relationship” (item 5), the *Agnew Relationship Measure: ARM-5* [14]. The other measure had already been identified in the original Delphi study (FABQ-W) [15].

For the remaining items (susceptible to placebo (item 3), patient has high trust and confidence in the practitioner (item 2), practitioner understanding of patient expectations and goals (item 7)) an appropriate Likert scale was constructed.

To maintain confidentiality and minimise bias, chiropractors’ and patients’ responses were kept unknown to each other by creating separate forms (See Additional File 1). Matching codes were placed on the patient and practitioner versions to pair responses.

The ability of the individual items on the questionnaire to fairly represent (content validity) the 18 predictors was assessed by a panel of chiropractors expert in SMT [16, 17]. The panel’s composition accorded with guideline recommendations, which state that content validity index (CVI) panels should comprise six to twelve participants with backgrounds representative of the target population [16, 17]. We recruited chiropractors with at least 7 years of experience using SMT as this matched the criteria used in the Delphi study for defining an expert.

Chiropractors known to the authors of this study were asked by email to suggest practitioners who had at least 7 years of clinical experience, and these were, in turn, approached by email to ask if they would like to join the panel. A panel of 6 experts was formed from responses to this invitation. The panel members were asked to assess the items in the questionnaires for clarity and relevance (e.g., to what degree do you think you understand this patient’s expectations and goals?) using four categories: not relevant; needs major revision; needs minor revision; and very relevant. Consistent with guideline recommendations, responses were dichotomised and assigned a value of ‘*one*’ for “needs minor revision” or “very relevant” and ‘*zero’* for “not relevant” or “needs major revision” categories [16, 17].

Panel members were not asked to assess the inventories (ARM-5, FABQ-W). An Item-Content Validity Index (I-CVI) was calculated for each remaining item by summing the values for each rater and then dividing by the number of raters. Based on previous research, an item was retained if its I-CVI was greater than 0.79 [16, 17]. Of the 16 predictor items created specifically for these two questionnaires, 3 recorded an I-CVI of 0.83 (items 4, 6, 18), and the remainder rated 1.0. Subsequently, all items were retained.

The panel was also asked to make recommendations that might improve the phrasing of the retained items and this resulted in some minor grammatical changes to improve the wording.

### Measures: final questionnaires (additional file 1)

#### The patient questionnaire

1. Patient has a history of good response to previous SMT (item 1).

This predictor item was assessed by the question, “How did you respond to previous manipulation for back pain”. A rating was sought on a Likert scale ranging between 0 and 10, where 0 was “very much worse”, 5 “no change” through to 10 “completely better”. The pilot study excluded new patients who had never experienced SMT for LBP.

2. Patient has high trust in practitioner (items 2 and 5).

The Agnew Relationship measure (ARM-5) is composed of a practitioner and patient’s form (each 5 items) and is designed to assess the client-therapist (item 2) and therapist-client (item 5) relationship. It is rated on a 7-point Likert scale ranging from “strongly disagree” (a score of 1) to strongly agree (a score of 7). Since we only recruited chiropractors, the word “therapist” was replaced with “chiropractor” in order to make it more situationally specific. One item is reverse coded (I have difficulty working as a joint partnership with my chiropractor/patient). Acceptable reliability and validity have been demonstrated with internal reliabilities of 0.77 and 0.87 [14].

3. Patient likely to respond to placebo (contextual factors) (item 3).

As we were concerned about the conceptual difficulties surrounding the word “placebo”, its various interpretations [18], its emotionally laden content and the impact of asking this question, a decision was made to replace it on the Likert scales with the less emotionally laden word “expectation”. Patients’ expectations have been identified as one of the major mechanisms contributing to contextual factors [19]. Thus, this predictor item was replaced with the question, “Please rate what you expected to happen to your back pain symptoms immediately after the low back manipulation”. The patients were again offered a Likert scale in order to rate their previous response to SMT between 0 and 10, where 0 was “a very great deal worse”, 5 “no change”, and 10 “A very great deal better”.

4. Patient has a comprehensive understanding of their condition (Item 4).

The question “How well do you understand your low back condition?” was used for this predictor item. A 0 to 10 Likert scale was offered with a scale ranging from 0 “I do not understand my back condition at all” to 10 “, I completely understand my low back pain”.

5. Chiropractor understanding of patient expectations and goals (Item 7).

To explore the possible bi-directional nature of this predictor item, we decided to ask both patient and practitioner the question, “To what degree did you (the patient/chiropractor) think the chiropractor understood your expectations and goals today”. Respondents were asked to record responses using a 5-point Likert scale that ranged from 0 (Strongly disagree), 3 (neutral), and 5 (Strongly agree).

6. Signs and Symptoms of the Low Back Pain (Items 9, 10, 11, 12, 13).

These predictor items are a cluster of the patient’s clinical information regarding how they experience their low back pain and are rated as “Yes” or “No”. These were; Have there been symptoms < 16 days? (item 9); Does pain improve with exercise but not rest? (item 10); Are there any symptoms in the lower extremities? (item 11); Does the patient has an acute condition of < 14 days? (item 12); Are there any symptoms distal to the knee? (item 13)

7. Fear-Avoidance Beliefs Subscale for Work (FABQ-W) (Item 17).

The FABQ-W, a subscale of the FABQ, seeks to understand a person’s degree of fear of work-related activities leading to avoidance behaviours. This questionnaire comprises 11 items seeking a response about how normal work affects or would affect participants’ back pain [15]. The questionnaire response options range from “Completely disagree” to “Completely agree”. Its reliability and validity have been extensively investigated, with a reported internal reliability of 0.75 [20].

8. Presence of an audible release (Item 18).

Response options to the question “Was there a production of a clicking sound (cavitation) at the moment of thrust?” included “Yes, easily heard”, “Yes, just heard”, “unsure”, and finally, “No”.

9. Actual response to SMT measure (outcome measure).

Patients were asked, “Please rate how your back symptoms actually changed immediately after the low back manipulation you received today?”. A Likert scale that ranged between 0 and 10, where 0 was “very much worse”, 5 “no change” through to 10 “completely better”, was used to record responses.

#### The chiropractor questionnaire (additional file 1)

Like the patient questionnaire, the chiropractor questionnaire also contained the ARM-5 (practitioner version) and the degree to which they perceived understanding the patient’s expectations and goals (same 11-point Likert scale).

1. Professional opinion of patient’s health status (Items 6 and 8).

The chiropractor was asked to rate the patient’s health status on a 7-item scale ranging from “Very poor”, to Unsure”, through to “Excellent”.

2. Changes in active and passive ranges of motion (Items 14 and 15).

Chiropractors were asked to provide the range of motion data by rating the amount of decreased of the “active range of motion” (item 14) and the “passive range of motion” (item 15) on a 4-option scale ranging from “No”, “Mild”, “Moderate”, and “Severe”.

3. Replication of patient’s symptoms (Item 16).

This section also sought a rating on the question, “Did the presence of spinal springing and/or end range loading closely reproduce the patient’s symptoms” (No, Yes, Unsure).

4. An open text box was placed at the end of the chiropractor’s questionnaire, asking for feedback on ways the questionnaire might be improved.

Two scales (Ask Understand Remember Assessment (Aura Scale) [21] and Patient-Doctor Relationship Questionnaire (PDRQ-9) [22]) were included in the data collection and measured patient communication self-efficacy and therapeutic alliance within a psychotherapy context (respectively) and formed part of the larger study but were not included in this pilot study.

### Methods for pilot testing the questionnaire

#### Sample size

There are various recommended sample sizes for a pilot study [23]. A common “rule of thumb” is that the ratio of subjects to predictors be at least 10:1 [24]. More recent research has suggested that it is better to have sample sizes of 100 for 5 predictor variables that are correlated with one another at a medium-level [25], and this study could potentially produce 5 predictor variables. Consequently, we sought to achieve a sample size of 100 by recruiting 10 chiropractors who would subsequently distribute the questionnaire to 10 consecutive patients who agreed to participate.

#### Recruitment

Convenience sampling was used to approach potential chiropractors who had used SMT for more than seven years. This time frame was selected as it was used for expert delineation in the original Delphi study. These practitioners were informed by email or telephone contact of the project by the student researcher and informed that they were free not to participate or withdraw at any stage without any consequences.Also, advertisements were placed on the Western Australian Chiropractors’ Facebook page seeking participants.

As chiropractors who agreed to participate were asked to recruit ten consecutive patients, all were informed of the patients’ inclusion criteria i.e., non-specific lower back pain (NS LBP) and that SMT would be used in their treatment session. New patients who had not experienced SMT for NS LBP were excluded.

#### Questionnaire implementation

Initial email and telephone contact with known chiropractors was made. If an expression of interest was returned, then the study information letter was forwarded. Once signed consent from the chiropractor was obtained, a package with 10 chiropractor and patient surveys was delivered to the practice. Instructions were given to have reception staff present the information letter to patients. If the patients met the inclusion criteria and agreed to participate, they were provided with a questionnaire to complete after their consultation. The chiropractor was also to complete their questionnaire at the end of the treatment session. A sealed ‘postal’ box was provided for sealed confidential questionnaires to be ‘delivered’. Instructions were also given to approach all patients (consecutively) with non-specific LBP once the study began and continued until the 10 patient questionnaires were completed.

Due to the straight forward nature of the required questionnaire, no onsite training was given to the chiropractor or clinic staff. Contact details of the researchers were provided on the information letter and the questionnaire should there be any questions or concerns.

### Data analysis

Data was entered and analysed in SPSS v.24 (IBM Corp, Armonk NY, USA) after being scanned for any incomplete or corrupt data. Double entry was not used. Descriptive statistics of the variables were generated.

The scales were investigated for the presence of floor and ceiling effects whereby no more than 15% of respondents should achieve the highest or lowest scores in a sample size of 50 or more people [26]. Frequencies, mean scores, modal scores and standard deviations were calculated for all items.

Finally, internal consistency (Cronbach’s alpha) levels for the ARM-5 and FABQ-W were calculated.

## Results

### Chiropractor and patient recruitment

Over a 3-week period, 23 chiropractors were approached via email and telephone to recruit the required 10 participants. It was not possible to determine why non-responding chiropractors refused to participate as they chose not to reply to telephone calls or emails.

The data collection took place in 2020 during the COVID-19 pandemic in Western Australia. This resulted in restrictions being imposed on chiropractic practices that involved, among others, periods of closure. There was regular email and telephone contact (2–4 weeks) with the participating practices to monitor data collection rates, recruitment ended after three months because of time constraints surrounding this project.

Of the 10 chiropractors who agreed to participate, 5 returned ten completed patient and practitioner questionnaires. Two chiropractors failed to reach the requested number, returning a further 13 questionnaires. Three chiropractors either did not respond to emails and phone calls or indicated that it was not possible to complete the study because of the impact of COVID restrictions. This resulted in a final patient sample size of 63 from 7 chiropractors.

### Patient responses

Patients’ responses to the items created specifically for this study demonstrated mean and median scores that approximated each other with small standard deviations (Table 2). These items also demonstrated ceiling effects (Additional File 2). This was most evident in item 7, which sought patients’ rating of their chiropractor’s level of understanding of their expectations and goals for the consultation in question.

**Table 2:** Patients’ and chiropractor scores on questionnaire items created to measure those who respond strongly and instantly to SMT for non-specific LBP (n = 63)

Predictor Item	Missing	Scale range	Mean (SD)	Median	Actual scale
Patient previous response to SMT (item 1)	13	0–10	8.4 (1.1)	8.0	6–10
Patient expected response today (item 3)	3	0–10	8.2 (1.4)	8.0	3–10
Patient actual change today (outcome measure)	3	0–10	8.5 (1.2)	8.5	4–10
Patient understands own LBP condition (item 4)	3	0–10	8.4 (1.2)	9.0	4–10
Patient thinks chiropractor understand their expectations (item 7)	4	0–10	8.9 (1.1)	10.0	6–10
F.A.B.Q-W (item 17)	17	0–42	12.13 (7.8)	12	7–35
ARM-5: Patient view of TA (item 2)	4	0–35	33.31 (2.3)	34	28–35
ARM-5: Chiropractor view of TA (item 5)	3	0–35	31.72 (3.3)	31	20–35

SMT: spinal manipulative therapy; LBP: low back pain; SD: standard deviation; TA: therapeutic alliance; FABQ-W; Fear Avoidance Beliefs Questionnaire work subscale; ARM:-5 Agnew Relationship Measure:

Internal reliability for the FABQ-W was good (Cronbach Alpha = 0.85), despite its poor patient completion. In contrast, only four patients and three chiropractors failed to complete the ARM-5; however, its internal reliability for both the practitioners (Cronbach Alpha = 0.66) and patients (Cronbach Alpha = 0.60) was poor [27, 28].

Nearly 80% of patient respondents reported either “just” or “easily” hearing the audible release of the SMT. Only 8% reported not hearing the audible release.

The patients’ responses to the presence of their NS LBP signs and symptoms indicated that the majority perceived themselves as experiencing a recent onset acute episode without referred pain (Table 3).

**Table 3:** Distribution of 63 patient responses for the signs and symptoms associated with their low back pain

Sign & Symptom reported by patient	Yes (%)	No (%)	Missing (%)
Duration of symptoms < 16 Days (item 9)	41 (65.1)	18 (28.6)	4 (6.3)
Pain improves with exercise, not rest (item 10)	34 (54.0)	26 (41.3)	3 (4.8)
Patient has acute condition (< 14 days) (item 11)	42 (66.7)	18 (28.6)	3 (4.8)
No symptoms in the lower extremities (item 12)	16 (25.4)	44 (69.8)	3 (4.8)
No symptoms distal to the knee (item 13)	5 (7.9)	55 (87.3)	3 (4.8)

#### Chiropractor responses

Chiropractors rated almost 60% of their patients’ health (item 6 and 8) as either “very good” or “excellent”. “Good” (23%), “Average” (16%) and “Poor” (3%) were much less common. Nearly two in every three patients were rated as having their pain reproduced with spinal springing or at end-range loading (item 16). A considerably smaller percentage of practitioners reported that the patient’s pain was not reproduced with spinal springing (22%), and only of them were “unsure” (8%).

Almost 80% of patients were rated by the chiropractor as having either mild or moderate decreases in their active and passive lumbar spine range of motion (Table 4).

**Table 4:** Practitioners assessment of decreased lumbar spine ranges of motion for the 63 patients

Signs & Symptoms	None (%)	Mild (%)	Moderate (%)	Severe (%)	Missing (%)
Decreased AROM (item 14)	4 (6.3)	37 (58.7)	14 (22.2)	4 (6.3)	4 (6.3)
Decreased PROM (item 15)	6 (9.5)	35 (55.6)	12 (19)	5 (7.9)	5 (7.9)

#### Chiropractors feedback (open-ended)

Three responses were recorded to the open-ended question. No comments were made in respect of ‘usability’ other than the positioning of the consent form on the survey. A change in wording was suggested for the cause of the presence of pain below the knee due to ambiguity surrounding whether the pain was due to local knee pathology or to referred pain. The final comment sought the inclusion of an additional question asking for the number of times the patient had consulted with the chiropractor, as it was thought to be an influencing factor.

## Discussion

This study sought to create an understandable questionnaire that was reliable and ‘usable’ for testing the 18 predictor items, based on a recent Delphi study [12], to predict those who would have a strong and instantaneous response to SMT. The development of the questionnaire necessitated that both practitioner and patient complete assessments of the SMT consultation so that measures for all 18 items could be created in accordance with the opinions of the experts in the Delphi study.

The ‘expert’ panel process to review the questionnaires’ clarity and relevance yielded few changes. Only minor feedback was obtained from the participating pilot study chiropractor suggesting that the questionnaire had demonstrated reasonable “usability”. However, the patient responses from the pilot study suggested that these are sensitive to bias and were not capturing the complexity and diversity of a therapeutic alliance as reflected in their levels of expectations.

Surprising, the TA (ARM-5) measure, which had previously demonstrated an acceptable internal reliability [28] failed to do so in our study. The reason for this remains unknown. In contrast, patients failed to ‘warm’ to the FABQ-W, and many did not complete it, despite demonstrating strong internal consistency. The most likely explanation was its length (11 items). Consequently, both instruments will need to be reviewed and replaced with more appropriate measures if a larger study is conducted. This will likely involve a shorter measure of fear avoidance in relation to work activities and another measure of TA.

Unfortunately, this post-graduate project was considerably impacted by the Western Australian COVID-19 restrictions, and this may have yielded an unclear picture of the likely difficulties of collecting patient data for a larger study. Also, it was constrained by university academic timelines required for submission. Thus, the data collection was halted after 3 months. At first glance, 10 consecutive patients with NSLBP would seem a very achievable number within a short timeframe. This was not the case. Further patient responses suggested that only patients with a strong positive opinion about their chiropractor and the use of SMT were likely to volunteer. Perhaps some form of financial incentive may need to be offered to obtain a sample size with sufficient diversity for analysis.

Demographic information was not collected for patients or chiropractors because it was not necessary for the objectives of this study but nonetheless may be confounding factors [29]. Also, due to time constraints, we did not form a focus group of patients who had received SMT for NS LBP to assess the questionnaire for clarity and relevance. This may have impacted on the reliability of the patient component of the questionnaires. For example, the item ‘presence of cavitation’ was structured into “Yes, easily heard/Yes, just heard /no/ unsure” categories. The creation of clearer definitions, especially for ‘unsure’, would be advisable in future studies to create a more reliable and valid measure.

The poor psychometric properties of the created items may reflect underlying conceptual difficulties. The comprehensive measuring of the factors, as suggested by practitioners in the Delphi study, such as contextual factors (placebo), will require instruments with better reliability and validity. Some of these measures were adapted from a psychological background (ARM-5 for TA) and may not be appropriate for a musculoskeletal injury context. Further investigations may need to explore this possibility.

Finally, it has been shown that identifying subgroups is no easy task and along with the findings of this study that the road ahead to create this questionnaire will involve considerable work yet [30].

### Lessons learned

The COVID pandemic added many layers of complexity to this student research project. The local COVID restrictions varied across the data collection period and created times where patient attendance to chiropractors was only recommended when a serious condition existed. While many aspects of a chiropractic program curriculum could be conducted via online avenues, active research projects exploring SMT that required recruitment were more severely impacted. Not only did they vary within very short time frames, but they also created an air of uncertainty for chiropractic practices and patients. This most likely played out poorly in that chiropractors and patients had many more important issues to deal with rather than volunteering for a study. Perhaps it is unsurprising that the patients who participated and valued chiropractic care enough to ‘brave COVID” returned heavily biased questionnaires.

It is difficult to think of alternative recruitment strategies. In retrospect, the solution most likely to produce better participation was to delay. However, there was considerable uncertainty about COVID restrictions going forward and the time limited nature of the graduate student played a major role in planning this project. Sufficient time had to be left for the data analyses and write up.

## Conclusion

A series of measures were created to quantify the 18 predictor items thought to best predict patients with low back pain who might experience an instant and strong positive response to SMT as identified by a panel of chiropractors with considerable SMT experience [12]. The questionnaire review process suggested good clarity and required only minor revisions. The pilot study encountered difficulties with chiropractor and patient recruitment. Finally, the data analysis revealed significant challenges with the measures chosen. Other measures need to be identified and explored before a fully powered study can be conducted.

### Electronic supplementary material

Below is the link to the electronic supplementary material.

Supplementary Material 1

Supplementary Material 2

## Data Availability

Deidentified data is available however, requests will require additional Ethics approval.

## List of Abbreviations

ARM-5: Agnew relationship measure
CI: Confidence intervals
CVI: Content Validity Index
FABQ-W: Fear Avoidance Beliefs Questionnaire (work subscale)
I-CVI: Item Content Validity Index
LBP: Low back pain
SMT: Spinal manipulation therapy
TA: Therapeutic alliance
