# Supplementary figures and images for: Creating and testing a questionnaire to predict immediate and strong positive responders to spinal manipulative therapy for non-specific low back pain. A pilot study
Source: Chiropr Man Therap. 2023 Sep 26;31:40. doi: 10.1186/s12998-023-00510-3 (PMC10523686; doi:10.1186/s12998-023-00510-3)

**Additional File 1.**

**Practitioner Questionnaire**


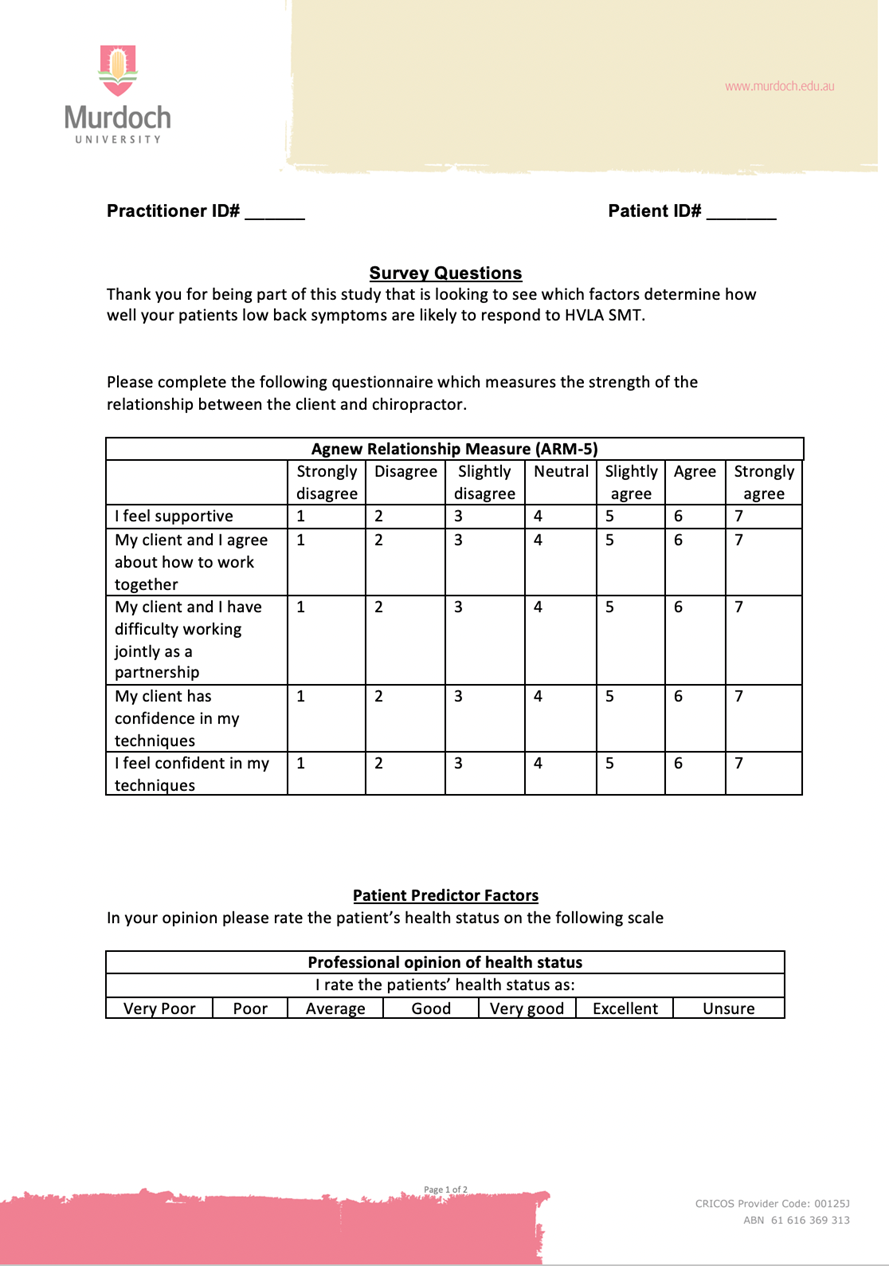


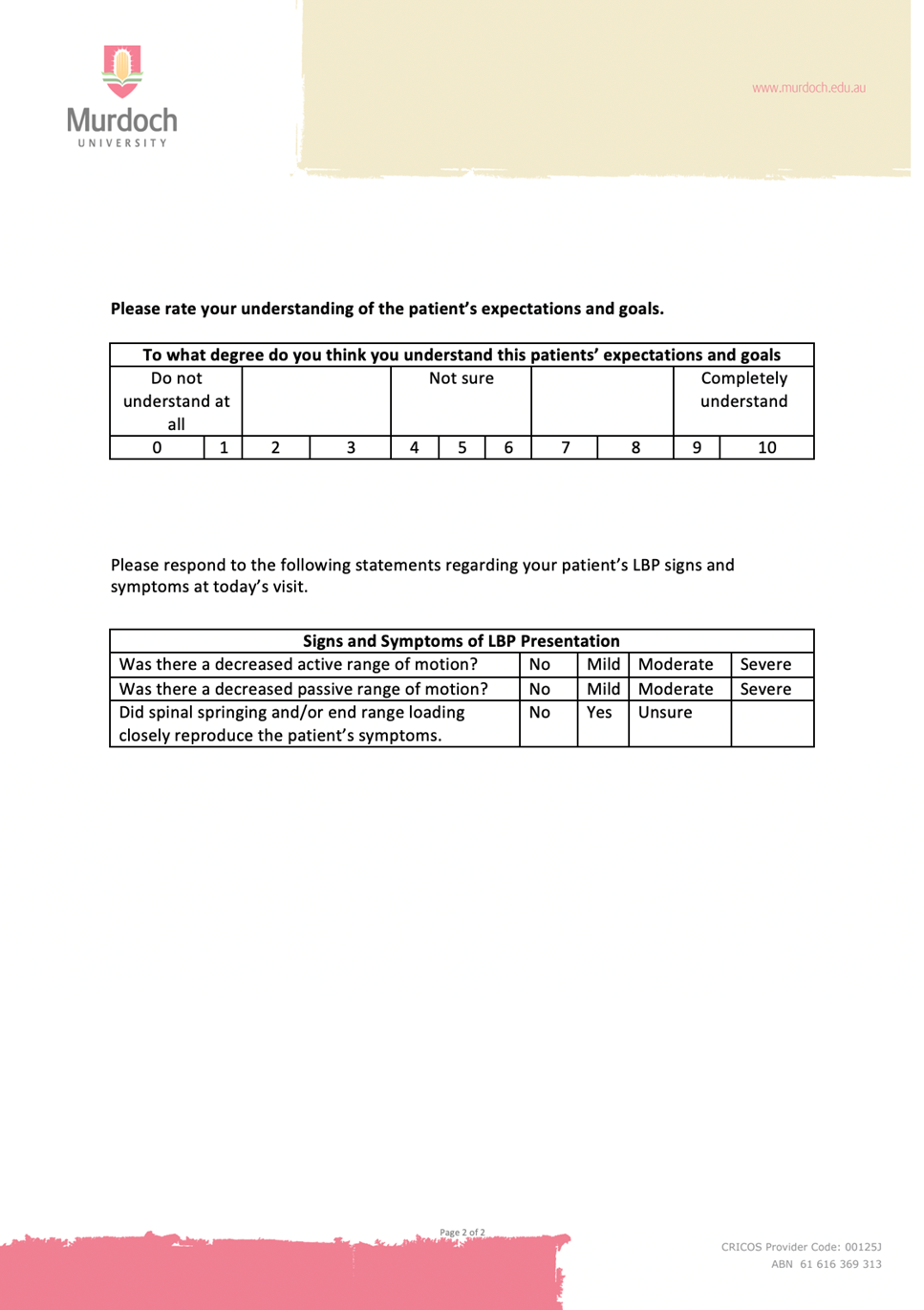


**Patient Questionnaire**


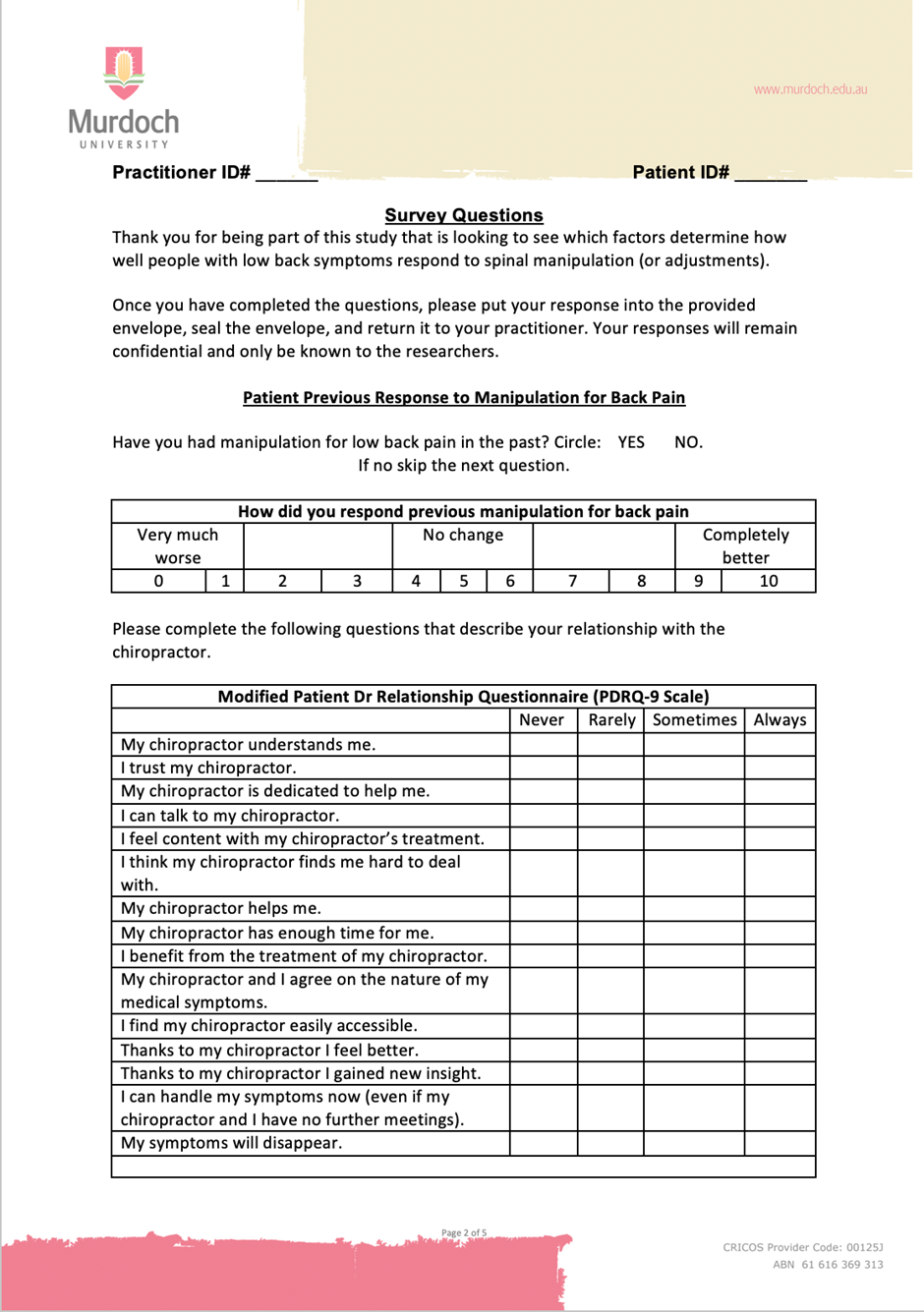


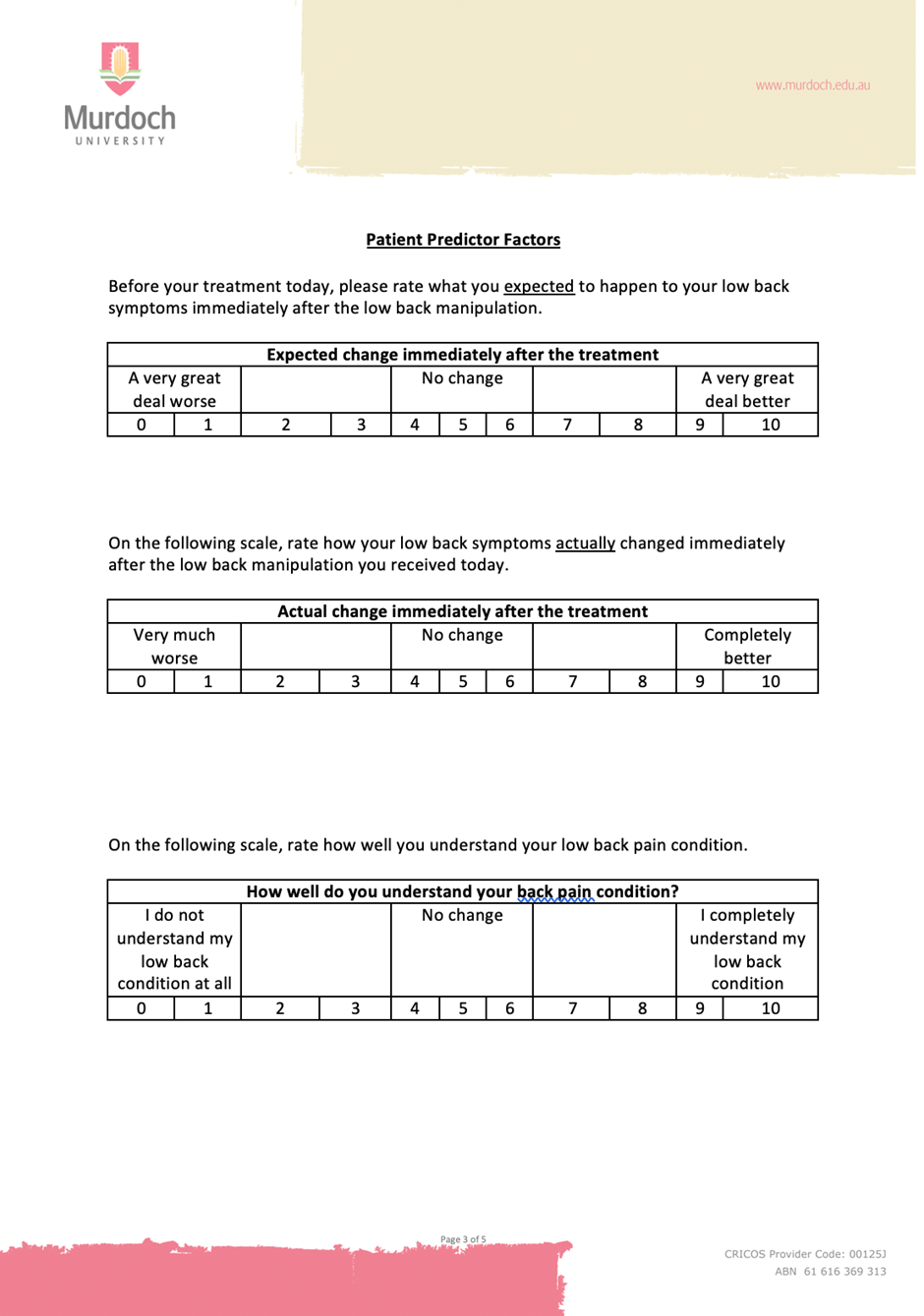


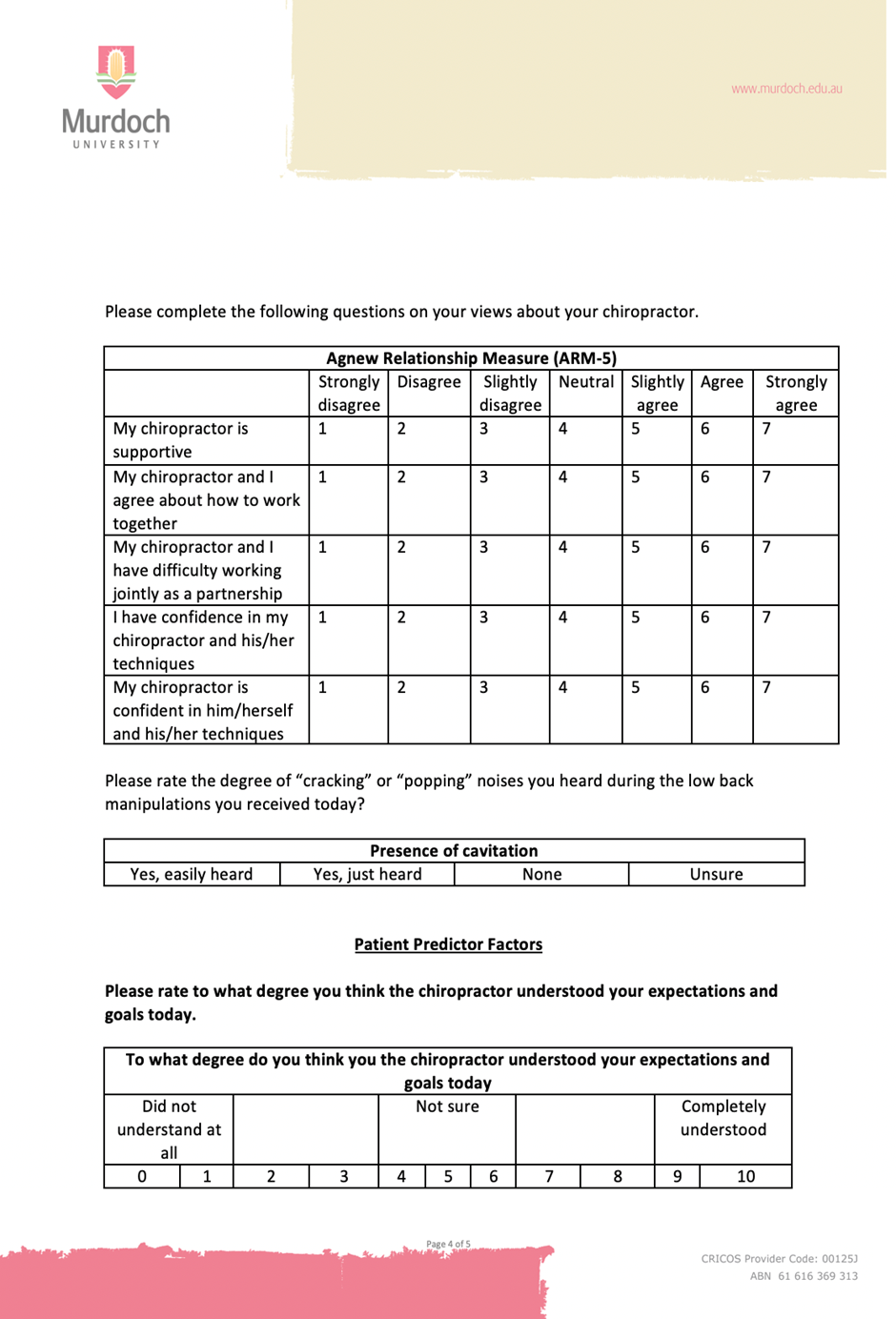


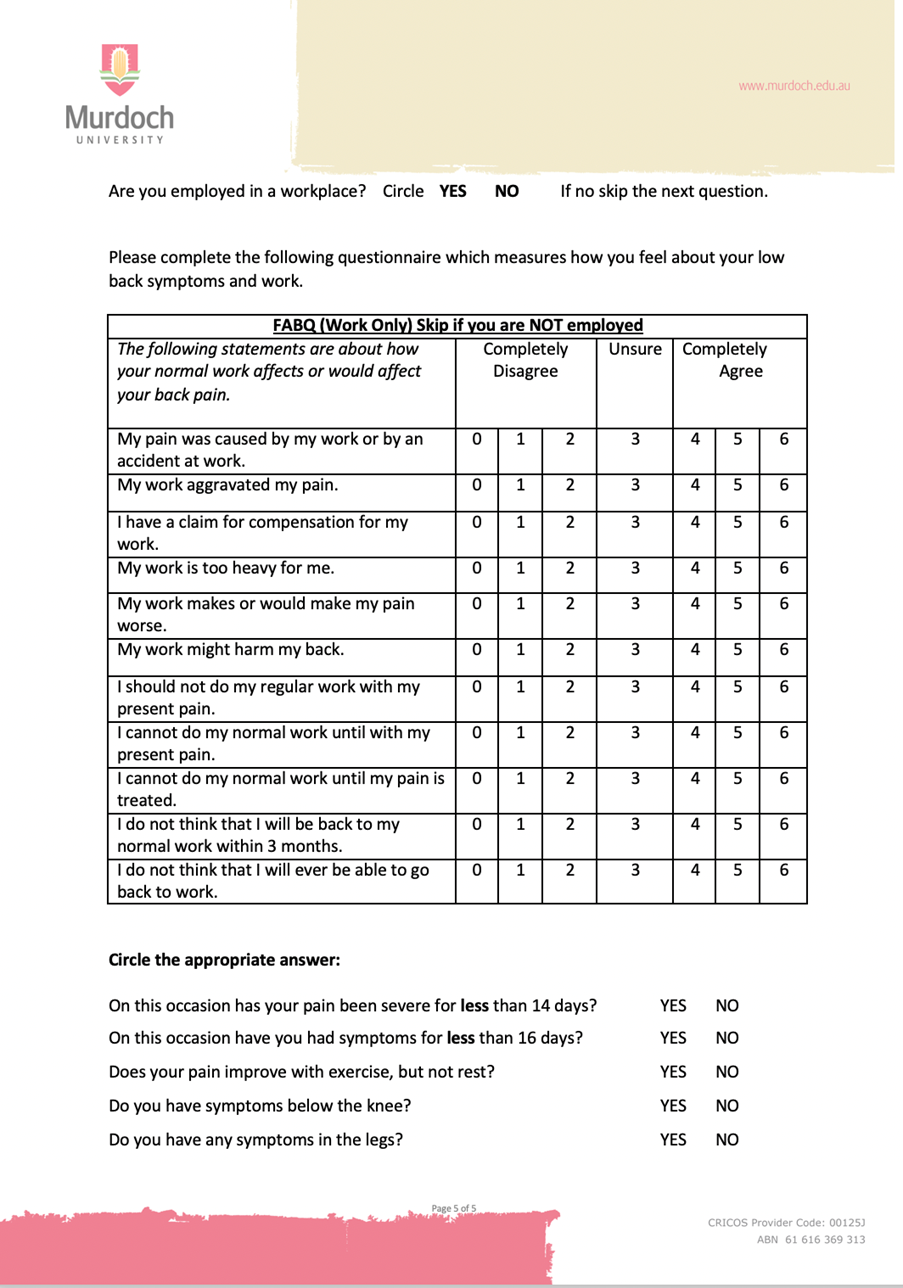

Supplement: Supplementary file 1 — Supplementary Material 1 [file 12998_2023_510_MOESM1_ESM.docx]
