# Supplementary material for: Creating and testing a questionnaire to predict immediate and strong positive responders to spinal manipulative therapy for non-specific low back pain. A pilot study
Source: Chiropr Man Therap. 2023 Sep 26;31:40. doi: 10.1186/s12998-023-00510-3 (PMC10523686; doi:10.1186/s12998-023-00510-3)
Supplement: Supplementary file 2 — Supplementary Material 2 [file 12998_2023_510_MOESM2_ESM.docx]

| *Patients’ response distribution on items created to measure response to SMT (n=63)* | | | | | | | | |
| --- | --- | --- | --- | --- | --- | --- | --- | --- |
| **Likert response** | **Missing** | **3**  **N (%)** | **4**  **N (%)** | **6**  **N (%)** | **7**  **N (%)** | **8**  **N (%)** | **9**  **N (%)** | **10**  **N (%)** |
| **Pt previous response to SMT** (item 1) | 13 |  |  | 2 (4) | 9 (19) | 16 (32) | 12 (24) | 11 (22) |
| **Pt expected response today** (item 3) | 3 | 1 (2) |  | 4 (7) | 14 (23) | 12 (20) | 18 (30) | 11 (18) |
| **Pt actual change today (outcome measure)** | 3 |  | 1 (2) |  | 12 (20) | 17 (28) | 14 (23) | 16 (27) |
| **Pt understands own LBP condition** (item 4) | 3 |  | 1 (2) | 1 (2) | 10 (17) | 17 (28) | 20 (33) | 11 (18) |
| **Pt thinks chiropractor understand their expectations** (item 7) | 4 |  |  | 2 (3) | 3 (5) | 7 (12) | 17 (29) | 30 (51) |
| **Chiro thinks understand Pt’s expectations** (item 7) | 5 |  |  |  | 8 (14) | 21 (36) | 13 (22) | 16 (28) |

*Note: Scale scores are missing the values “!”, “2”, and “5” is missing as these were not selected by participants. Abbreviations: SMT= spinal manipulative therapy. LBP= low back pain*
